# Clinical decision-making before discharge in hospitalized persons with schizophrenia: a Spanish Delphi expert consensus

**DOI:** 10.3389/fpsyt.2024.1412637

**Published:** 2024-06-10

**Authors:** José Manuel Montes, Luis Agüera-Ortiz, Anna Mané, Jose Martinez-Raga, Luis Gutiérrez-Rojas

**Affiliations:** ^1^ Psychiatry Department, Hospital Universitario Ramón y Cajal, Universidad de Alcalá, Madrid, Spain; ^2^ Centro de Investigación en Red de Salud Mental, CIBERSAM, Instituto de Salud Carlos III, Madrid, Spain; ^3^ Department of Psychiatry, Instituto de Investigación Sanitaria (imas12), Hospital Universitario 12 de Octubre, Madrid, Spain; ^4^ Psychiatry Department, Parc de Salut Mar, Barcelona, Spain; Hospital del Mar Medical Research Institute (IMIM), Barcelona, Spain; ^5^ Psychiatry Department, Hospital Universitario Doctor Peset & Universitat de Valencia, Valencia, Spain; ^6^ Department of Psychiatry, University of Granada, Granada, Spain; ^7^ Psychiatry Department, Hospital Clínico San Cecilio, Granada, Spain

**Keywords:** schizophrenia, inpatient, hospitalization, discharge, expert consensus, Delphi method

## Abstract

**Introduction:**

The care of people with schizophrenia (PWS) is usually provided in an outpatient setting by community mental health teams. However, PWS frequently require inpatient treatment because of a wide array of clinical, personal and/or social situations. Unfortunately, to our knowledge, there are no guidelines available to help psychiatrists in the decision-making process on hospital discharge for PWS. The aim of this project was to develop an expert consensus on discharge criteria for PWS after their stay in an acute inpatient psychiatric unit.

**Methods:**

Using a modified Delphi method a group of 42 psychiatrists throughout Spain evaluated four areas of interest regarding this issue: clinical symptomatology, treatment-related factors, follow-up health care units after discharge, and physical health and monitoring.

**Results:**

After two rounds, among the 64 statements, a consensus was reached for 59 (92.2%) statements. In three (17.7%) of the 17 statements on ‘clinical symptomatology’ and 2 (13.3%) of the 15 statements on ‘follow-up health care units after discharge’, a consensus was not reached; in contrast, a consensus was reached for all statements concerning ‘treatment-related factors’ and those concerning ‘physical health and monitoring’. The consensus results highlight the importance for discharge of the control of symptoms rather than their suppression during admission and of tolerability in the selection of anantipsychotic.

**Discussion:**

Although there is a lack of relevant data for guiding the discharge of PWS after hospitalization in an acute inpatient psychiatric unit, we expect that this consensus based on expert opinion may help clinicians to take appropriate decisions.

## 1 Introduction

Schizophrenia is a chronic and disabling disorder characterized by a complex symptomatology involving positive, negative and cognitive symptoms and a chronic and recurrent course of disease (1, 2). Schizophrenia is associated with significant health, social, occupational, and economic burdens as a result of its early onset and severe and often persistent symptoms (3). Although the care of people with schizophrenia (PWS) is usually provided in an outpatient setting by community mental health teams, these individuals frequently require inpatient treatment as well. Indeed, one in two individuals with a first episode of psychosis (FEP) will require at least one hospitalization within 7 years after their first contact with mental health services (4). Furthermore, hospitalization is considered unavoidable for PWS who exhibit an acute exacerbation that cannot be managed safely on an outpatient basis (5).

Treatment plans for PWS should include achieving and maintaining recovery, maximizing quality of life and adaptive functioning, and reducing or eliminating symptoms. However, only a small proportion of PWS achieve recovery, with proportions ranging from 14% to 24% when recovery is defined on the basis of clinical and functional remission (6, 7). While full recovery is possible for PWS, it is not an attainable goal after acute treatment and cannot be the driver of hospital discharge. In addition, and importantly, the reasons behind a psychiatric inpatient hospitalization could involve a wide array of clinical, personal and/or social situations, including but not limited to the following: threat to oneself or others; hallucinations directing harm to oneself or others where there is a risk of the patient taking action on them; acute disordered/bizarre behavior; psychomotor agitation or retardation; cognitive impairment that interferes with activities of daily living; and outpatient psychiatric treatment failure so that the patient requires professional observation (8, 9). Therefore, once a PWS has been hospitalized, the criteria for discharge should be based on the assessment of all these variables. Unfortunately, to our knowledge, there are no guidelines available to help psychiatrists in the decision-making process on hospital discharge for PWS.

The aim of this project was to develop an expert consensus on discharge criteria for PWS after their stay in an acute inpatient psychiatric unit (AIPU).

## 2 Materials and methods

### 2.1 Study design

This study was conducted using a modified Delphi method. When there is no evidence, the evidence is controversial, or the evidence cannot be obtained through epidemiological or experimental research, recommendations can be gathered from expert opinion using a systematic approach such as the Delphi method (10, 11). The aim of the Delphi method is to reach an agreement on a specific topic. This method has several important characteristics, such as anonymity to avoid dominance, iteration to allow participants to change their opinion, controlled feedback of the aggregate responses and participants’ individual responses, and summary measures that allow the quantification of the degree of consensus (10). The method has been widely used in psychiatry research for a variety of purposes, such as making estimations where there is incomplete evidence, making predictions, and more commonly determining collective values or defining foundational concepts (11).

The project took place in five steps: aim definition, expert selection, questionnaire development, Delphi rounds, statistical analysis and reporting/dissemination. These steps are briefly described below.

The project did not involve patient participation. Owing to its nature, following the general Spanish regulations on biomedical research (that is, Law 14/2007, of July 3, on Biomedical Research), this project did not require the evaluation of an Ethics Committee.

### 2.2 Definition of aims

The project coordinator (JMM) was responsible for the study conceptualization with the abovementioned aim and for selecting another 4 experts (LAO, AMS, JMR, and LGR) who made up the scientific committee.

### 2.3 Expert selection and development of the questionnaire

The scientific committee selected 37 psychiatrists throughout Spain with a wide geographic representation; selected psychiatrists had to have experience in the management of acute psychosis and with clinical activity in an AIPU. All experts had at least 4 years of experience in the management of patients with acute psychosis, including their management in the acute inpatient unit.

Each member of the scientific committee individually provided their individual proposal for the statements to be included in the questionnaire based on the literature and their experience with the criteria and requirements for the discharge of a PWS admitted to an AIPU. The scientific committee discussed all the proposed statements and agreed on the final questionnaire. Additionally, five members of the scientific committee answered the questions in the questionnaire (42 participants total). The final questionnaire comprised 64 statements that were grouped into four areas of interest: clinical symptomatology (17 statements); treatment-related factors (18 statements); follow-up health care units after discharge (15 statements); and physical health and monitoring (14 statements). The specific questions are shown in **Tables 1**–**4** when presenting the results. All statements were rated using a 9-point Likert scale of agreement, where 1 meant “fully disagree” and 9 meant “fully agree”. In addition, every statement included a free-text field for recording potential comments from the participants.

**Table 1:** Clinical symptomatology.

Question/Issue	Round	Likert-scale score									% C	M	% OR
		1 Fully Disagree	2	3 Fairly Disagree	4	5 Neither Agree nor Disagree	6	7 Fairly Agree	8	9 Fully Agree			
**1.1** In a patient with a first psychotic episode, for discharge from the acute unit to home, in all cases is the disappearance of hallucinations necessary?	2	14.3 (14.3)	11.9 (19.0)	52.4 (26.2)	–	2.4 (2.4)	4.8 (7.1)	11.9 (16.7)	2.4 (11.9)	– (2.4)	78.6 (59.5)	3 (3)	21.4 (40.5)
**1.2** In a patient diagnosed with schizophrenia, for discharge from the acute unit to home, in all cases is the disappearance of hallucinations necessary?	2	19 (19.0)	19 (23.8)	45.2 (21.4)	4.8 (4.8)	2.4 (2.4)	2.4 (9.5)	4.8 (14.3)	2.4 (4.8)	– (–)	83.2 (64.2)	3 (3)	16.8 (35.8)
**1.3** In a patient with a first psychotic episode, the persistence of auditory hallucinations does not preclude discharge from the acute unit to home care if there is an affective and behavioral distancing of the patient from the hallucinations.	1	–	–	4.8	2.4	–	4.8	28.6	47.6	11.9	88.1	8	11.9
**1.4** In a patient diagnosed with schizophrenia, the persistence of auditory hallucinations does not preclude discharge from the acute unit to home care if there is an affective and behavioral distancing of the patient from the hallucinations.	1	–	–	–	4.8	–	–	14.3	42.9	38.1	95.3	8	4.8
**1.5** In a patient diagnosed with schizophrenia, the persistence of nonauditory hallucinations does not preclude discharge from the acute unit to home care if there is an affective and behavioral distancing of the patient from the hallucinations.	1	–	–	4.8	–	–	–	19	52.4	23.8	95.2	8	4.8
**1.6** In a patient diagnosed with schizophrenia, the persistence of chronic auditory hallucinations—even with a behavioral response to them—does not preclude discharge from the acute unit to home if the hallucinations do not pose a risk to the integrity of others or to the patient themselves.	1	–	–	7.1	–	2.4	9.5	52.4	19	9.5	80.9	7	19
**1.7** In a patient diagnosed with schizophrenia, to proceed to discharge from the acute unit to the home, a patient’s critique of the delusional ideation is necessary.	2	2.4 (9.5)	9.5 (19.0)	57.1 (31.0)	11.9 (4.8)	7.1 (9.5)	7.1 (9.5)	4.8 (16.7)	– (–)	– (–)	69 (59.5)	3 (3)	31 (40.5)
**1.9** In a patient with a first psychotic episode, the persistence of the delusional ideation that motivated the admission does not preclude discharge from the acute unit to home if there is an affective and behavioral distancing from the delusional ideation.	1	–	–	14.3	2.4	2.4	9.5	40.5	26.2	4.8	71.5	7	28.5
**1.8** In a patient diagnosed with chronic schizophrenia, the persistence of the delusional ideation that motivated the admission does not preclude discharge from the acute unit to home if there is an affective and behavioral distancing from the delusional ideation.	1	–	–	–	2.4	2.4	–	31	50	14.3	95.3	8	4.8
**1.10** In a patient diagnosed with schizophrenia, an organization of thought and behavior that allows the patient to perform activities of daily living is a necessary condition for discharge from the acute unit to home.	2	– (–)	2.4 (7.1)	7.1 (11.9)	– (4.8)	9.5 (7.1)	7.1 (2.4)	52.4 (33.3)	14.3 (21.4)	7.1 (11.9)	73.8 (66.6)	7 (7)	26.2 (33.4)
**1.11** In a patient with a first psychotic episode, an organization of thought and behavior that allows the patient to perform activities of daily living is a necessary condition for discharge from the acute unit to home.	1	–	4.8	9.5	–	9.5	2.4	16.7	40.5	16.7	73.9	8	26.1
**1.12** In a patient diagnosed with schizophrenia, a patient’s distancing from the suicidal ideation that motivated the admission is a sufficient criterion for discharge from the acute unit to home.	2	– (4.8)	4.8 (4.8)	28.6 (28.6)	2.4 (2.4)	19 (16.7)	7.1 (9.5)	14.3 (9.5)	9.5 (9.5)	14.3 (14.3)	38.1 (33.3)	5 (5)	61.9 (66.7)
**1.13** In a patient diagnosed with schizophrenia, to discharge from the acute unit to home, the patient’s criticism of the suicidal ideation that motivated the admission is a necessary criterion.	1	7.1	–	14.3	–	2.4	7.1	26.2	16.7	26.2	69.1	7	31
**1.14** In a patient diagnosed with schizophrenia, if there is a personal history of a serious suicide attempt, discharge from the acute unit to home is possible only if there is patient criticism of suicidal ideation.	1	4.8	–	4.8	2.4	7.1	2.4	23.8	21.4	33.3	78.5	8	21.4
**1.15** The absence of significant improvement in the cognitive symptomatology of a patient diagnosed with schizophrenia contraindicates discharge from the acute unit to home only if the patient is unable to perform instrumental activities of daily living.	2	– (4.8)	4.8 (9.5)	16.7 (16.7)	– (2.4)	14.3 (9.5)	4.8 (7.1)	47.6 (33.3)	9.5 (11.9)	2.4 (4.8)	59.5 (50.0)	7 (6.5)	40.5 (50.0)
**1.16** The absence of significant improvement in the patient’s negative symptomatology contraindicates discharge from the acute unit to home only if the patient is unable to perform basic activities of daily living (self-care).	2	2.4 (4.8)	2.4 (7.1)	11.9 (16.7)	4.8 (4.8)	14.3 (14.3)	16.7 (9.5)	35.7 (23.8)	7.1 (11.9)	4.8 (7.1)	47.6 (42.8)	6 (6)	52.5 (57.2)
**1.17** In a patient diagnosed with schizophrenia, if there is ideation or heteroaggressive behavior on admission, discharge from the acute unit to home is possible only if there is patient criticism of that ideation/behavior.	2	– (–)	2.4 (2.4)	9.5 (9.5)	2.4 (11.9)	2.4 (2.4)	11.9 (14.3)	40.5 (26.2)	23.8 (19.0)	7.1 (14.3)	71.4 (59.5)	7 (7)	28.6 (40.5)

% C, proportion of consensus; M, median; % OR, out of region.

The shaded area indicates the presence and location of the consensus site.

For the statements evaluated in the second round, results of the first round appear between brackets."-", It means 0.0%.

**Table 2:** Treatment-related factors.

Question/Issue	Round	Likert-scale score									% C	M	% OR
		1 Fully Disagree.	2	3 Fairly Disagree	4	5 Neither Agree nor Disagree	6	7 Fairly Agree	8	9 Fully Agree			
**2.1** The patient with schizophrenia should be discharged with the same treatment that was effective during admission.	1	2.4	–	–	–	–	–	26.2	40.5	31	97.7	8	2.4
**2.2** The antipsychotic treatment at the discharge of a patient with schizophrenia should reduce the impact of his or her symptoms to avoid the associated risks that caused and led to his or her admission.	1	–	–	–	–	–	–	2.4	50	47.6	100	8	0
**2.3** The patient with schizophrenia should be discharged with the same doses of antipsychotic treatment that he or she received in the days prior to discharge.	1	2.4	2.4	9.5	4.8	–	9.5	23.8	33.3	14.3	71.4	7	28.6
**2.4** The antipsychotic treatment at the discharge of a patient with schizophrenia should not worsen negative symptoms.	1	–	–	4.8	7.1	4.8	14.3	14.3	28.6	26.2	69.1	8	31
**2.5** The antipsychotic treatment at the discharge of a patient with schizophrenia should not worsen affective symptoms.	1	–	–	–	–	2.4	4.8	31	35.7	26.2	92.9	8	7.1
**2.6** The antipsychotic treatment at the discharge of a patient with schizophrenia should not worsen cognitive symptoms.	1	–	–	4.8	2.4	2.4	9.5	21.4	31	28.6	81	8	19
**2.7** The patient with schizophrenia needs to receive an antipsychotic treatment at discharge that has shown a good tolerability profile in the days prior to discharge.	1	–	–	2.4	–	2.4	2.4	9.5	38.1	45.2	92.8	8	7.2
**2.8** The antipsychotic treatment at the discharge of a patient with schizophrenia should have an adequate tolerability profile in the medium to long term.	1	–	–	–	–	–	7.1	9.5	31	52.4	92.9	9	7.1
**2.9** The patient with schizophrenia should not be discharged with antipsychotic treatment that leads to a considerable increase in physical morbidity.	1	–	–	4.8	–	2.4	7.1	23.8	33.3	28.6	85.7	8	14.3
**2.10** Prior to discharge, every patient with schizophrenia should have received information about the reasons for his or her treatment and the goals to be achieved with it.	1	–	–	–	–	2.4	2.4	7.1	31	57.1	95.2	9	4.8
**2.11** The patient with schizophrenia should be discharged with a treatment that allows good adherence to it in the medium to long term.	1	–	–	–	–	–	–	7.1	45.2	47.6	100	8	0
**2.12** The patient with schizophrenia, before being discharged from an AIPU, must be committed to treatment and recognize it as a way to achieve an improvement in his or her clinical situation.	2	– (–)	– (2.4)	4.8 (7.1)	– (–)	7.1 (9.5)	14.3 (16.7)	42.9 (28.6)	26.2 (23.8)	4.8 (11.9)	75.7 (64.3)	7 (7)	23.8 (35.7)
**2.13** If the reason for the relapse that led to admission in a patient with schizophrenia is a lack of adherence, treatment with long-acting injectable antipsychotics should be attempted in the acute unit.	1	–	2.4	–	–	4.8	2.4	16.7	38.1	35.7	90.4	8	9.5
**2.14** Complex doses such as several doses per day should be avoided in the antipsychotic treatment at discharge.	1	–	–	–	–	–	2.4	11.9	38.1	47.6	97.6	8	2.4
**2.15** Antipsychotic polytherapy (= 2 antipsychotics at full dose or ≥3 antipsychotics at any dose) should be avoided in the antipsychotic treatment at discharge.	1	–	2.4	2.4	–	4.8	9.5	33.3	19	28.6	80.9	7	19
**2.16** If the patient with schizophrenia presents dual pathology, the antipsychotic treatment at discharge should be oriented to this profile.	1	–	–	–	–	2.4	4.8	16.7	50	26.2	92.9	8	7.1
**2.17** If the patient with schizophrenia presents dual pathology, specific pharmacological treatment for the addictive disorder should be included at discharge.	1	–	–	2.4	4.8	2.4	11.9	28.6	26.2	23.8	78.6	7.5	21.4
**2.18** If the patient with schizophrenia presents symptoms resistant to two or more antipsychotics, a trial with clozapine should be performed during admission if there is no contraindication and it has not previously failed.	1	–	–	–	–	7.1	4.8	7.1	40.5	40.5	88.1	8	11.9

% C, proportion of consensus; M, median; % OR, out of region.

For the statements evaluated in the second round, results of the first round appear between brackets.The shaded area indicates the presence and location of the consensus site."-", It means 0.0%.

**Table 3:** Health care and other resources at and after discharge.

Question/Issue	Round	Likert-scale score									% C	M	% OR
		1 Fully Disagree.	2	3 Fairly Disagree	4	5 Neither Agree nor Disagree	6	7 Fairly Agree	8	9 Fully Agree			
**3.1** Coordination with discharge destination resources is needed.	1	–	–	–	–	–	–	7.1	23.8	69	100	9	0
**3.2** The discharge report must include the appointment at the resource to which the patient is referred.	1	–	–	–	–	2.4	2.4	4.8	7.1	83.3	95.2	9	4.8
**3.3** An appointment with the patient’s psychiatrist must be scheduled within 7 days of discharge from the hospital.	1	–	–	9.5	–	7.1	7.1	26.2	26.2	23.8	76.2	7.5	23.8
**3.4** An appointment with the patient’s psychiatrist must be scheduled within 14 days of discharge from the hospital.	1	2.4	–	9.5	2.4	7.1	4.8	14.3	19	40.5	73.8	8	26.2
**3.5** An appointment with the patient’s psychiatrist must be scheduled within 21 days of discharge from the hospital.	1	19	16.7	33.3	2.4	4.8	9.5	–	7.1	7.1	69	3	31
**3.6** The pharmacological treatment must be specified in the discharge report together with the prescription.	1	–	–	–	–	–	–	–	4.8	96.2	100	9	0
**3.7** All the information about the assistance and follow-up at discharge must be written in the discharge report in a detailed and understandable way for the patient.	1	–	–	2.4	–	–	2.4	7.1	11.9	76.2	95.2	9	4.8
**3.8** Regardless of the resource at discharge, the patient should be assigned a multidisciplinary mental health team of reference and have the contact information.	1	–	–	–	–	–	4.8	4.8	19	71.4	95.2	9	4.8
**3.9** An individualized treatment and rehabilitation care plan should be prepared for each patient prior to discharge.	2	– (2.4)	– (–)	7.1 (9.5)	– (–)	7.1 (9.5)	4.8 (11.9)	45.2 (23.8)	16.7 (21.4)	19 (21.4)	80.9 (66.6)	7 (7)	19 (33.4)
**3.10** Social assessment of the patient with schizophrenia is essential prior to discharge from the hospital.	2	– (–)	– (2.4)	2.4 (14.3)	– (2.4)	16.7 (14.3)	– (9.5)	54.8 (26.2)	16.7 (14.3)	9.5 (16.7)	81 (57.2)	7 (7)	19 (42.8)
**3.11** The participation in patient advocacy groups should be contemplated during admission.	2	7.1 (7.1)	2.4 (4.8)	16.7 (19.0)	14.3 (7.1)	33.3 (35.7)	4.8 (7.1)	14.3 (14.3)	4.8 (2.4)	2.4 (2.4)	21.5 (19.1)	5 (5)	78.5 (81.9)
**3.12** The entire health care network, including psychosocial resources, should have access to the shared electronic health record and other information tools to facilitate the continuity of care.	2	– (–)	2.4 (4.8)	14.3 (14.3)	2.4 (4.8)	11.9 (14.3)	7.1 (4.8)	28.6 (21.4)	11.9 (14.3)	21.4 (21.4)	61.9 (57.1)	7 (7)	38.1 (22.9)
**3.13** The involvement of the family/caregivers should be promoted during admission.	1	–	–	–	–	–	9.5	16.7	40.5	33.3	90.5	8	9.5
**3.14** Postdischarge housing and its appropriateness based on previous discharge status and patient characteristics (including suicide risk) should be carefully assessed prior to discharge.	1	–	–	4.8	2.4	–	2.4	7.1	33.3	50	90.4	8.5	8.5
**3.15** Patients with dual pathology at discharge should also be referred to specific addiction resources.	1	–	4.8	–	–	2.4	4.8	16.7	38.1	33.3	88.1	8	11.9

% C, proportion of consensus; M, median; % OR, out of region.

For the statements evaluated in the second round, results of the first round appear between brackets.The shaded area indicates the presence and location of the consensus site."-", It means 0.0%.

**Table 4:** Physical health and monitoring.

Question/Issue	Round	Likert-scale score									% C	M	% OR
		1 Fully Disagree.	2	3 Fairly Disagree	4	5 Neither Agree nor Disagree	6	7 Fairly Agree	8	9 Fully Agree			
**4.1** All patients with a first psychotic episode should have a neuroimaging test prior to discharge.	1	2.4	–	7.1	–	2.4	4.8	11.9	23.8	47.6	83.3	8	16.7
**4.2** All patients with schizophrenia should have a blood count prior to discharge.	1	–	–	–	4.8	4.8	2.4	9.5	21.4	57.1	88	9	11.9
**4.3** All patients with schizophrenia should have a thyroid hormone assay prior to discharge.	2	– (–)	2.4 (–)	2.4 (7.1)	– (4.8)	9.5 (11.9)	14.3 (9.5)	11.9 (11.9)	16.7 (14.3)	42.9 (40.5)	71.5 (66.7)	8 (8)	28.6 (33.3)
**4.4** All patients with schizophrenia should have a prolactin level assay prior to discharge.	2	– (–)	2.4 (2.4)	4.8 (11.9)	2.4 (4.8)	11.9 (11.9)	7.1 (7.1)	35.7 (28.6)	23.8 (16.7)	11.9 (16.7)	71.4 (62)	7 (7)	28.6 (38)
**4.5** In all patients with schizophrenia, glucose, cholesterol and triglyceride levels should be measured prior to discharge.	1	–	–	–	2.4	4.8	–	11.9	23.8	57.1	92.8	9	7.2
**4.6** In all patients with schizophrenia an ECG should be performed during admission.	1	–	–	2.4	2.4	4.8	4.8	21.4	31	33.3	85.7	8	14.3
**4.7** In all patients with schizophrenia, liver function should be studied before discharge.	1	–	–	–	2.4	9.5	–	21.4	21.4	45.2	88	8	11.9
**4.8** In all patients with schizophrenia, renal function should be studied before discharge.	1	–	–	–	4.8	7.1	4.8	21.4	19	42.9	83.3	8	16.7
**4.9** In the case that a patient with schizophrenia presents associated smoking, antitobacco advice should be given, and treatment should be offered to treat such an addiction before discharge.	1	–	2.4	4.8	2.4	2.4	9.5	26.2	23.8	28.6	78.6	8	21.4
**4.10** All patients with schizophrenia should be screened for sexually transmitted diseases (HIV, LUES) before discharge.	2	– (–)	– (–)	11.9 (19.0)	2.4 (–)	7.1 (7.1)	7.1 (9.5)	42.9 (28.6)	19 (21.4)	9.5 (14.3)	71.4 (64.3)	7 (7)	28.6 (35.7)
**4.11** All patients with schizophrenia should be screened for hepatitis B and C virus prior to discharge.	2	– (–)	– (–)	9.5 (16.7)	– (–)	7.1 (11.9)	4.8 (9.5)	47.6 (23.8)	19 (21.4)	14.3 (16.7)	80.9 (61.9)	7 (7)	21.4 (39.1)
**4.12** All patients with schizophrenia should undergo a toxicology screen in urine preferably at the beginning of the admission period.	1	–	2.4	7.1	–	4.8	2.4	19	16.7	47.6	83.3	8	16.7
**4.13** In all patients with schizophrenia of childbearing age, a pregnancy test should be performed prior to discharge and preferably at the beginning of the admission period.	1	2.4	2.4	4.8	–	11.9	9.5	14.3	14.3	40.5	69.1	8	31
**4.14** All patients with schizophrenia should be evaluated for the presence of extrapyramidal symptoms during the admission period.	1	–	–	–	–	–	2.4	7.1	23.8	66.7	97.6	9	2.4

% C, proportion of consensus; M, median; % OR, out of region.

For the statements evaluated in the second round, results of the first round appear between brackets.The shaded area indicates the presence and location of the consensus site."-", It means 0.0%.

### 2.4 The Delphi rounds

All participants were informed of the objectives of the project, and completing the questionnaire meant that they consented to participate. We administered the questionnaire in two rounds via a specific website for the study. For the second round, we included only statements for which no consensus had been reached in the first round. In this second round, when accessing the questionnaire, the participants were informed of the overall results of the first round using a bar graph with the frequency distribution of the answers on the Likert scale and the comments on those statements provided by all participants. The participants were also provided their answers to the first round and were asked to rate the statement again.

### 2.5 Statistical analysis

The analysis was essentially descriptive, and the relative frequencies of the responses on the 9-point Likert scale were used. We considered that a consensus was reached when the median was located in the agreement (i.e., scores 7 to 9 on the Likert scale) or disagreement (i.e., scores 1 to 3 on the Likert scale) area and at least two-thirds of the responses were located within those areas. The median was also used as a measure of the strength of agreement. In addition, we calculated the mean, the proportion of consensus and the proportion of responses out of the region of consensus.

All analyses were performed using MS Excel.

## 3 Results

### 3.1 Overall results

Forty-two participating psychiatrists (37 selected psychiatrists plus 5 members of the scientific committee) completed the two rounds of the Delphi. After two rounds, among the 64 statements, a consensus was reached for 59 (92.2%) statements. In three (17.7%) of the 17 statements on ‘clinical symptomatology’ and 2 (13.3%) of the 15 statements on ‘follow-up health care units after discharge’, a consensus was not reached; in contrast, a consensus was reached for all statements concerning ‘treatment-related factors’ and those concerning ‘physical health and monitoring’.

### 3.2 Clinical symptomatology

Regarding hallucinations, there was a consensus against the statement that the disappearance of hallucinations should be mandatory at discharge in patients with an FEP (median 3) or with schizophrenia (median 3) (**Table 1**). In contrast, there was a consensus that discharge would be possible when auditory hallucinations persist in patients with an FEP or PWS or when nonauditory hallucinations persist in a PWS, providing the patient shows affective and behavioral distancing from them (median 8 in all cases). There was also a consensus that even if chronic auditory hallucinations persist, they do not preclude discharge if they do not pose a risk for the integrity of the patient or others (median 7). A similar consensus was reached for the persistence of delusional ideation, which, according to the respondents, does not preclude discharge provided that the patient shows distancing from them, both in patients with an FEP* (median 7) and in those with schizophrenia (median 8).

When proceeding with discharge, a consensus was reached that an organization of thought and behavior that allows the performance of activities of daily living are needed both for patients with FEP* (median 8) and for those with schizophrenia (median 7). If suicidal ideation was the reason for admission, there was no consensus that the patient showing distancing from it was enough for discharge (median 5); instead, there was a consensus that, for a discharge, the patient’s criticism of suicidal ideation was needed if that was the reason for admission (median 7), as was his or her personal history of a serious suicide attempt (median 8).

There was no consensus on whether the absence of a significant improvement in cognitive symptoms in a patient unable to perform instrumental activities of daily living (median 7) or the absence of a significant improvement in negative symptoms in a patient unable to perform basic daily life activities (median 6) was a contraindication for discharge.

If there was heteroaggressive ideation or behavior upon admission, there was a consensus that discharge from the acute unit is possible if the patient shows criticism of that ideation/behavior (median 7).

### 3.3 Treatment-related factors 

There was a consensus that patients should be discharged with the same antipsychotic (median 8) at the same dose (median 7) that was administered in the days prior to discharge and that the antipsychotic should reduce the impact of the symptoms that led to admission (median 8) (**Table 2**).

There was also a consensus that the antipsychotic prescribed at discharge should not worsen negative, affective, or cognitive symptoms (median 8 for the three statements). The respondents also agreed that the antipsychotic prescribed at discharge should have shown a good tolerability profile in the days prior to discharge (median 8), should have an adequate tolerability profile in the medium and long term (median 9), and should not increase the physical comorbidity to a relevant extent (median 8).

The respondents agreed with several issues related to treatment adherence, including that the patients should receive information on the reasons behind the prescription of the antipsychotic and the treatment goals (median 9), the patient should be committed to treatment and recognize that it leads to an improvement in her or his clinical situation (median 7) and the antipsychotic prescribed should allow good adherence in the medium and long-term (median 8). In addition, if the reason for admission was related to the lack of adherence, treatment with a long-acting antipsychotic should be considered (median 8). A consensus was also reached on avoiding an antipsychotic regime with complex dosing (median 8) and on avoiding antipsychotic polypharmacy (median 7).

In patients with a concurrent substance use disorder (i.e., dual disorders), the antipsychotic should be oriented to that profile (median 8), and treatment of the substance use disorder should be included at discharge (median 7.5). If the patient has shown symptoms resistant to 2 or more antipsychotics, there was consensus that a trial with clozapine should be performed during admission, provided that it is not contraindicated and there is no history of previous failure to clozapine (median 8).

### 3.4 Health care and other resources at and after discharge

The respondents agreed that coordination with follow-up resources is needed and that an appointment with the outpatient psychiatrist should be scheduled within 7 (median 7.5) or 14 days (median 8) after discharge; there was also a consensus against scheduling the appointment with the outpatient psychiatrist within 21 days after discharge (median 3) (**Table 3**). There was a consensus that the discharge report should include the appointment with the resource where the patient has been referred (median 9), the prescribed pharmacologic treatment (median 9), and all the information about the assistance and monitoring at discharge (median 9). Furthermore, there was agreement that, regardless of to which resource the patient is referred, the patient should be assigned to a mental health team (median 9), individualized treatment and rehabilitation plans should be prepared before discharge (median 7) and a social assessment is essential prior to discharge (median 87).

There was no agreement on the involvement of patient advocacy groups during admission, but there was agreement that the involvement of family/caregivers should be promoted (median 8). There was no consensus that the entire health care network should have access to electronic health records and other information tools to facilitate continuity of care.

A consensus was reached that postdischarge housing should be carefully assessed before discharge (median 8.5). The respondents also agreed that patients with a concurrent substance use disorder should be referred to an addiction unit (median 8).

### 3.5 Physical health and monitoring

There was agreement on all issues posed to respondents (**Table 4**). Thus, there was consensus on performing all of the following laboratory or ancillary assessments prior to discharge (**Table 5**): neuroimaging (median 8), complete blood count (median 9), thyroid hormone blood test (median 8), prolactin level (median 7), fasting blood glucose and lipid determinations (median 9), liver (median 8) and renal function (median 8) tests and an EKG (median 8). In patients who use tobacco, tobacco cessation advice and treatment should be offered (median 8). There was also a consensus to perform screening for sexually transmitted diseases (median 7), hepatitis B and C (median 7), drug use (median 8), pregnancy in PWS of childbearing age (median 8), and the presence of extrapyramidal symptoms (median 9).

**Table 5:** Laboratory and other ancillary assessments recommended to be performed prior to discharge for hospitalized patients with schizophrenia.

Assessment	Median in the consensus
Neuroimaging	8
Complete blood count	9
Thyroid hormone blood test	8
Prolactin level assay	7
Fasting blood glucose and lipid determinations	9
Liver function	8
Renal function	8
EKG	8
Tobacco cessation advice and treatment	8
Screening for sexually transmitted diseases	7
Screening for hepatitis B and C	7
Urine toxicology screen	8
Pregnancy test in PWS of childbearing age	8
Presence of extrapyramidal symptoms	9

EKG, electrocardiogram; PWS, people with schizophrenia.

## 4 Discussion

In this consensus, using a modified Delphi method, a group of experts agreed on most of the issues regarding the criteria for hospital discharge and management in PWS after admission to an AIPU.

For both patients with an FEP and those with schizophrenia, the respondents agreed that for discharge, the disappearance of hallucinations is not needed. Instead, in the case of auditory or nonauditory hallucinations, the participants considered that for discharge, showing affective and behavioral distancing from hallucinations is enough. It is important to bear in mind that in approximately one-third of patients with schizophrenia, positive symptoms, such as hallucinations, persist despite adequate trials of antipsychotic medications (12). Moreover, even in the presence of chronic auditory hallucinations that have a behavioral impact, according to the respondents, they do not preclude discharge from an AIPU provided that they do not pose a threat to the patients or to others. However, in the case of nonauditory hallucinations, especially in the case of visual hallucinations, the presence of organicity and, therefore, the presence of a different primary psychotic disorder or the presence of a medical or neurological illness should be ruled out (13). The respondents also agreed against the idea that in a patient with delusional ideation, it is necessary that the patient show criticism of it. The respondents considered that the presence of affective and behavioral distancing from delusional ideation is enough for proceeding with discharge. For both patients with an FEP and those with schizophrenia, the respondents considered the condition necessary for discharge to be that a patient show an organization of thought and behavior that allows them to perform activities of daily living.

Compared with the general population, PWS are at high risk of suicide (14), with estimated lifetime rates ranging between 4% and 13% (15). Moreover, suicide has been reported to be the greatest relative risk factor for mortality in individuals with schizophrenia (16). Furthermore, it is estimated that the lifetime incidence of suicidal ideation among PWS is 34.5% (17). When suicide ideation was the reason that motivated admission, the respondents did not agree with the statement that distancing from suicidal ideation was enough to discharge the patient from the AIPU and that it was necessary for the patient to show criticism of suicidal ideation (18). It is likely that many psychiatrists believe that it is necessary to achieve remission or distance from the psychotic or affective symptoms that were the basis of suicidal ideation. Importantly, rates of suicide appear to increase within the first seven days after discharge (19); therefore, after discharge, it is important to achieve adequate family involvement and linkage to outpatient follow-up resources and/or those specific for suicide prevention. Similarly to suicide (20), there was a consensus that in cases of heteroaggressive ideation or behavior, it is necessary for patients to criticize that ideation or behavior to proceed with discharge.

The lack of a consensus on the need for significant improvement in cognitive or negative symptoms that allows individuals to perform activities of daily living is consistent with the fact that it is hardly realistic to expect a significant improvement during such a short period of time, bearing in mind the stability of those deficits during the course of the disorder (21) and the limited response of cognitive or negative symptoms to an antipsychotic treatment (22).

There was a consensus on all the statements on treatment-related factors. Thus, the respondents considered that patients should be discharged with the same antipsychotic at the same dose that was prescribed during the last days of the inpatient stay, although for the latter statement, the strength of the consensus was the weakest in this area of interest (median 7; with 14% of respondents showing disagreement). It is possible that this degree of disagreement could be related to the need to reduce the dose to improve tolerability. The statement with the highest strength of agreement was that the antipsychotic prescribed at discharge should have an adequate tolerability profile in the medium and long term (median 9). Clinical practice guidelines recommend the use of second-generation antipsychotics, especially those associated with a lower risk of weight gain, hyperprolactinemia, sexual dysfunction, or increased sedation (23). There was a consensus that antipsychotic treatment at discharge should not worsen affective, negative or cognitive symptoms. As mentioned above, patients with negative and cognitive symptoms exhibit a poorer response to antipsychotic treatment (17), and these symptoms have an important impact on functioning (24). Therefore, it is necessary to avoid the negative impact of antipsychotic treatment on these domains. Although the management of affective, negative or cognitive symptoms is still an unmet need in the treatment of PWS, it is generally accepted that second-generation antipsychotics are preferred, and due to their distinct pharmacological profile, there could be some differences among drugs in their effects on those symptoms (25–27). The respondents also agreed on all statements related directly or indirectly to treatment adherence, including the importance of informing the patient on treatment goals and commitment to their treatment and the importance of tolerability and treatment complexity. If poor adherence was the reason for relapse and admission, respondents also agreed that treatment with long-acting injectable antipsychotics should be attempted in the AIPU. In addition to poor illness insight, younger age is a risk factor for nonadherence to antipsychotic medication (28), and it is suggested that tolerability could be especially important in young adults, such as those experiencing an FEP. Overall, clinicians should look for antipsychotics that are adequately managed (i.e., dose escalation) to be able to control the symptoms of schizophrenia in the AIPU and show a good tolerability profile, especially in the medium and long term. The respondents also agreed on the importance of addressing concurrent substance use disorders both in the selection of antipsychotic medication and in the treatment of that disorder at discharge. Substance use disorder is a predictor of admission in patients with an FEP (29, 30), and integrated management of dual disorders improves outcomes (31).

There was consensus on the importance of good coordination between the acute care unit and health care resources after discharge. For such purposes, the information included in the discharge report is important and should include the pharmacologic treatments at discharge, the appointment of the patient with the health care resource, and the information on follow-up care after discharge. The appointment with the outpatient psychiatrist should take place soon after discharge, within 7 or 14 days; making an appointment after a longer interval following discharge (i.e., 21 days) was rejected by the experts. There was also agreement that individualized treatment and rehabilitation care plans should be prepared before discharge, although the strength of the consensus was weaker than that for other statements (median 7); it is possible that some respondents considered that these plans should be prepared by the outpatient mental health team of reference.

There was no consensus on the involvement of patient advocacy groups during admission. Importantly, although the available evidence is very limited, there is no evidence that the inclusion of peer support in patients with schizophrenia or other serious mental illness improves outcomes compared with standard treatment (32). In contrast, the experts agreed on promoting the involvement of family/caregivers during admission. There was also agreement that in patients with dual pathology, the patient should also be referred to a specific addiction health care resource.

The respondent agreed with all the statements regarding physical health monitoring. This consensus was generally consistent with the recommendations of the American Psychiatric Association (APA) for physical and laboratory assessments for patients with schizophrenia (3). However, in contrast to our consensus, the APA recommends performing brain imaging, drug toxicology screening and prolactin level testing if clinically indicated. We recommend that all patients with an FEP undergo neuroimaging. This recommendation is consistent with other expert recommendations (33). However, the authors of a recent systematic review did not find evidence to support the routine performance of neuroimaging testing in people with an FEP without associated neurological or cognitive impairment (34). This group of experts advocates performing a urine drug screening in all patients with an FEP or schizophrenia due to the implications of substance use on clinical outcomes and, especially, on the risk of relapse/readmission. While the APA guidelines state that this procedure should be considered only if clinically indicated, in patients with an FEP, this procedure is recommended in some guidelines (35). Regarding the determination of prolactin levels, the strength of the consensus was the weakest for physical monitoring (median 7). While the APA guidelines recommend monitoring prolactin levels only when clinically indicated (3), other guidelines suggest that baseline prolactin levels should be measured routinely if a patient is starting antipsychotic therapy and that a reassessment should be performed after three months of treatment if there are symptoms of hyperprolactinemia or if the patient is receiving an antipsychotic with known prolactin-elevating properties (23). It is important to consider that hyperprolactinemia is related to a wide array of health problems, such as galactorrhea, oligomenorrhoea and amenorrhea, impaired ovulation, sexual dysfunction, reduced bone mineral density and cardiovascular disease (36). In addition, although with somewhat mixed results (37), recent observational studies suggest a potential association between antipsychotic-induced hyperprolactinemia and an increased risk of occurrence of breast cancer, which led to some authors to recommend monitoring prolactin concentrations and avoiding the use of prolactin-elevating antipsychotics in women (38, 39).

Our study has several limitations. The expert consensus provides the lowest level of evidence. A threshold for consensus was established for two-thirds of the respondents. Although there is no a general agreement on the threshold to establish a consensus, other studies have used a more stringent threshold (i.e., 70%, 75%, or 80%). Finally, this is a Spanish national consensus, and thus, our recommendations cannot be generalized to other countries without proper adaptation.

In conclusion, although there is a lack of relevant data for guiding the discharge of PWS after hospitalization in an AIPU, we believe that this consensus based on expert opinion may help clinicians to make these decisions. The consensus highlights the importance of the control of symptoms rather than their suppression during admission and of tolerability in the selection of an antipsychotic. In fact, the selection of antipsychotic medication during the hospital stay should take into account not only its short-term effect but also the circumstances involving outpatient treatment, which patients will likely need after discharge.

## Data availability statement

The raw data supporting the conclusions of this article will be made available by the authors, without undue reservation.

## Author contributions

JM: Writing – review & editing, Writing – original draft, Visualization, Methodology, Investigation, Formal analysis, Conceptualization. LA: Writing – review & editing, Writing – original draft, Visualization, Methodology, Investigation, Formal analysis, Conceptualization. AM: Writing – review & editing, Writing – original draft, Visualization, Methodology, Investigation, Formal analysis, Conceptualization. JM: Writing – review & editing, Writing – original draft, Visualization, Methodology, Investigation, Formal analysis, Conceptualization. LG: Writing – review & editing, Writing – original draft, Visualization, Methodology, Investigation, Formal analysis, Conceptualization.
